# Prevalence and Risk Factors for Diabetic Peripheral Neuropathy in Type 2 Diabetic Patients From 14 Countries: Estimates of the INTERPRET-DD Study

**DOI:** 10.3389/fpubh.2020.534372

**Published:** 2020-10-20

**Authors:** Yanhui Lu, Pengbo Xing, Xue Cai, Dan Luo, Ruxue Li, Cathy Lloyd, Norman Sartorius, Mingzi Li

**Affiliations:** ^1^School of Nursing, Peking University Health Science Center, Beijing, China; ^2^Faculty of Wellbeing, Education and Language Studies, The Open University, Milton Keynes, United Kingdom; ^3^Association for the Improvement of Mental Health Programmes (AMH), Geneva, Switzerland

**Keywords:** diabetic peripheral neuropathy, prevalence, risk factors, depressive symptoms, diabetes mellitus

## Abstract

**Aim:** Diabetic peripheral neuropathy (DPN) is a common, severe microvascular complication of diabetes. Our study was to assess prevalence and risk factors for DPN in subjects with type 2 diabetes from 14 different countries.

**Methods:** A total of 2,733 subjects with type 2 diabetes aged 18–65 years (45.3% men, mean duration of diabetes = 8.8 years) were included to perform this International Prevalence and Treatment of Diabetes and Depression (INTERPRET-DD) study in 14 countries. After a structured questionnaire was used in face-to-face interviews to collect sociodemographic characteristics and medical records of the participating subjects, laboratory tests were carried out for clinical measurement. Depressive symptoms were diagnosed and measured using the Patient Health Questionnaire-9. The potential risk factors for DPN were determined by multilevel mixed-effects logistic regression, accounting for clustering of participants within the country. Robustness of the estimates was assessed by sensitivity analysis.

**Results:** The overall prevalence of DPN across different countries was 26.71%, whereas country-specific prevalences showed considerable variation. Multivariate analysis revealed that duration of diabetes (OR: 1.08 per 1-year increase, 95% CI: 1.06–1.09), poor glycemic control (OR: 1.11 per 1% increase in HbA1c, 95% CI: 1.05–1.18), and history of hypertension (OR: 1.58, 95% CI: 1.18–2.12), cardiovascular disease (OR: 2.07, 95% CI: 1.55–2.78) and depressive symptoms (OR: 1.92, 95% CI: 1.43–2.58) were independently and positively associated with the risk of DPN. Sensitivity analyses including or excluding patients from countries with extreme low or high prevalence of DPN yielded similar estimates in terms of trend and magnitude.

**Conclusions:** This international study illustrates that more than a quarter of individuals with type 2 diabetes developed DPN. The prevalence was positively associated with the duration of diabetes, poor glycemic control, and history of hypertension, cardiovascular disease and depressive symptoms.

## Introduction

Diabetes mellitus is a major public health issue. An estimated 451 million people were diagnosed with diabetes worldwide in 2017, and the number will increase to 693 million by 2045 (1). Compared with other chronic diseases, diabetes causes greater mortality, morbidity, disability and financial loss due to its complications (2). Diabetic peripheral neuropathy (DPN) is one of the most common and severe microvascular complications of diabetes (3). As a major contributor to peripheral nerve injury (4), DPN impairs quality of life and potentiates risks of disability in diabetic subjects (5). However, the prevalence and the risk factors of DPN on a global scale, especially in low- and middle-income countries, remain unclear.

Previous studies showed a fairly wide range: 8–75%, for the prevalence of DPN in diabetic patients (6–11). Likely, the heterogeneity in anthropometry, culture and ethnicity might have been confounded to these considerable disparities. Many epidemiological studies have examined clinical correlates of DPN (6, 7, 10, 12, 13). Apart from vascular risk factors (14), depressive symptoms have also been implicated in the pathogenesis of DPN (15). However, the prevalence and risk factors for diabetes-associated DPN have not been systematically investigated in adults of multiple countries.

The International Prevalence and Treatment of Diabetes and Depression Study (INTERPRET-DD) was conducted in 14 countries under the auspices of the Dialogue on Diabetes and depression (http://diabetesanddepression.org/). The study was aimed to investigate the prevalence and treatment of depression in type 2 diabetic patients (16). Thus, this study has provided us with an unprecedented opportunity to investigate the prevalence and risk factors of DPN as a common and severe complication of type 2 diabetes in multiple countries with different socio-economic development and cultural settings.

## Methods

### Study Population

The INTERPRET-DD study is an international study conducted between 2013 and 2015 in 14 countries including Argentina, Bangladesh, China, Germany, India, Italy, Kenya, Mexico, Pakistan, Poland, Russia, Serbia, Uganda, Ukraine. A detailed description of the research design and data collection procedure of the INTERPRET-DD study has been published previously (17). Briefly, a sample of consecutive outpatient clinic attenders with type 2 diabetes mellitus (T2DM) at each of the study sites were invited by the treating physician or diabetologist in the diabetes clinics to participate in the study between September 2013 and May 2015, with the purpose of enrolling a total of 200 T2DM patients in each country. The collaborating sites recruited the teams of investigators from leading centers of excellence in each country, with at least one psychiatrist and endocrinologist included. Diabetes clinics were based in either secondary or tertiary care centers, depending on the facilities available in each country. T2DM patients aged 18–65 years and diagnosed at least 12 months before enrollment were eligible study participants. The exclusion criteria were: diagnosis of T2DM <12 months; diagnosis of type 1 diabetes; inability to complete the survey tools because of communication or cognitive difficulties; any life-threatening or serious conditions (e.g., cancer, stroke in the last 6 months); being currently admitted or planning an admission for inpatient care to a hospital (unless admitted for diabetes self-management); pregnant women or women who gave birth within the last 6 months and clinically diagnosis of dependency on alcohol or other substances (not tobacco) or a diagnosis of schizophrenia. A total of 2,783 individuals with T2DM agreed to participate in the study, at a response rate of 92.3%. Response rates differed among countries, ranging from 64.7% (Ukraine) to 100% in Uganda, Mexico and India. However, only 2,733 participants were included in the final analysis, with 50 participants being excluded due to incomplete records. Institutional review board approval was obtained for each center in each country prior to protocol implementation. Written informed consent was obtained from all participants.

### Data Collection

Investigators from each site in each country completed an information form for each eligible individual. This form included information from medical records, such as age, duration of diabetes, family history of diabetes and presence/history of diabetes complications, which included cardiovascular disease, peripheral neuropathy, retinopathy, renal disease, peripheral vascular disease as well as associated disorders. Most recent measurements of blood pressure, height and weight, and HbA1c were also recorded. Participants were asked if they lived in what they considered to be a rural or an urban area, and reported their highest level of education (higher education was defined as any college, postgraduate or professional training). Marital status was defined as married/cohabiting vs. being single/widowed/divorced. Participants were also asked if they considered that they had a regular income. DPN was assessed clinically when participants showed symptoms of sensory abnormalities, tingling, numbness, burning pain with paresthesia in both lower extremities or pain of distal extremities lasting for more than 3 months through neurophysiological study, which was recorded in the medical records according to standard criteria (18). Body mass index (BMI) was calculated as weight in kilograms divided by height in meters squared. Hypertension was defined as self-reported history of hypertension, taking antihypertensive drugs, systolic BP ≥ 140 mmHg or diastolic BP ≥ 90 mmHg. The Patient Health Questionnaire-9 (PHQ-9), a self-report measure of depression symptoms based on the American Psychiatric Association's DSM-IV criteria for the diagnosis of a depressive episode, was used to identify probable depression (19). The presence of moderate/severe depressive symptomatology was defined as PHQ-9 scores > 9 according to a previous study (17). All questionnaires were completed with standard self-complete methods in appropriate languages, or assisted one-to-one collection. Where no existing translation/cultural adaption of the questionnaire was available, it was adapted using standard forward/back translation procedures. Moreover, questionnaires were ensured to be culturally applicable by each country's investigators through development of several iterative stages, including discussion and testing by a range of healthcare professionals and T2DM patients and concentrating on the meaning of terms as well as language.

### Statistical Analysis

All analyses were performed using Stata version 15.1 (StataCorp LP, College Station, TX, USA). A two-sided *P*-value of < 0.05 was considered statistically significant.

### Prevalence of DPN

We first estimated the overall and country-specific prevalences of DPN. Participants were categorized into six age groups: 18–39, 40–45, 46–50, 56–60 and 61–65 years old. The gender-, age- and residence-specific prevalences were also calculated.

### Cross-Sectional Analysis

Continuous variables were shown as means (standard deviation, SD) and categorical variables were presented as frequencies and percentages. Student *t*-test and χ2 test were used to compare continuous and categorical variables between participants with and without DPN, respectively. Multilevel mixed-effects logistic regression accounting for clustering of participants within the same country (meqrlogit procedure in Stata) was used to determine significant risk factors by putting all the covariates in the model. The multilevel model had explanatory variables at two levels: country and individual. Associations were reported as odds ratios (ORs) and 95% confidence intervals (CI). In addition, we performed a sensitivity analysis to minimize the presence of a potential bias by excluding participants from countries with extreme low or high prevalence of DPN.

## Results

A total of 2,733 participant aged 18–65 years were included in estimating the overall prevalence of DPN. The demographic profile of the study population stratified by countries is shown in Supplementary Table 1. A slightly lower proportion of men than women participated in the study (45.41%), ranging from 23.63% (Russia) to 63.79% (Germany). The majority of participants (72.5%) were married or cohabiting, ranging from 58.13% (Mexico) to 86.08% (China). The 69.0% participants had higher education, ranging from 42.71% (Russia) to 91.81% (Kenya). Only 16.7% participants had no regular income, ranging from 1.51% (Russia) to 47.78% (Mexico).

**Figure 1:**
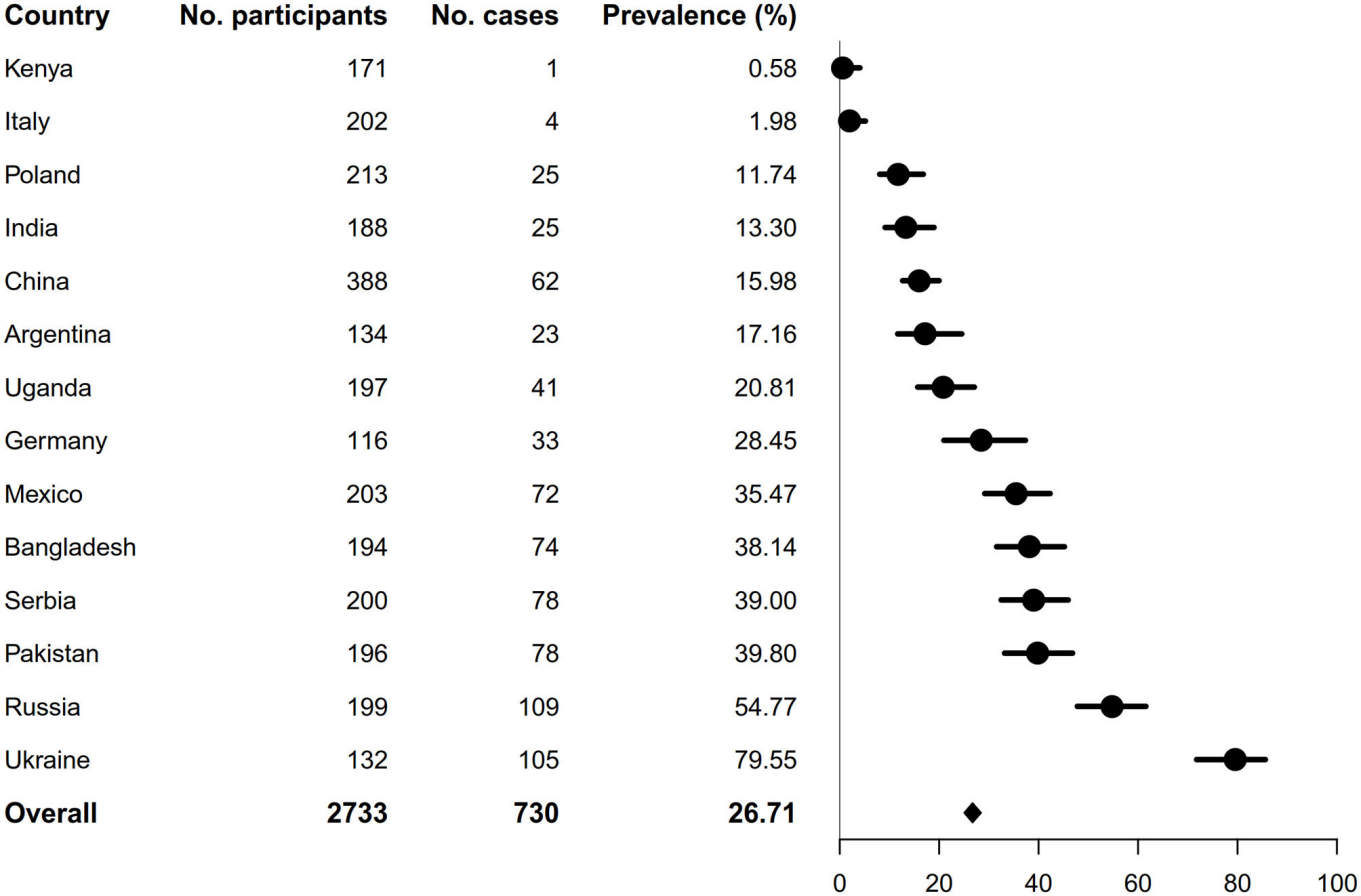
Prevalence of diabetic neuropathy by country.

**Figure 2:**
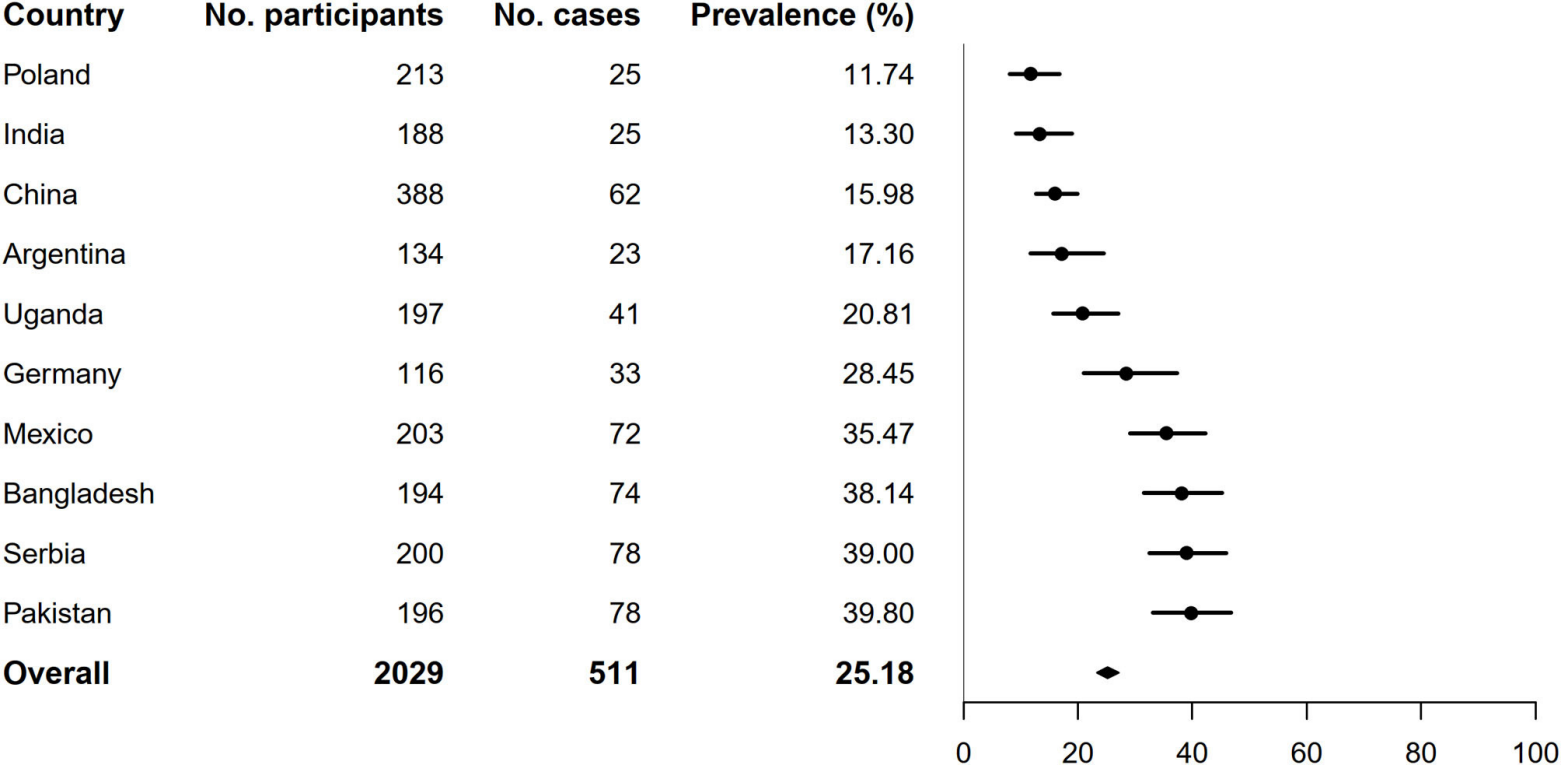
Prevalence of diabetic neuropathy by country after excluding participants from Kenya, Italy, Ukraine and Russia.

As shown in Figure 1, the overall prevalence of DPN was 26.71% (95% CI: 25.08–28.40). There was a significant difference in the prevalence of DPN across different countries (Figure 1). The participants from Kenya and Italy had the lowest prevalence of DPN (0.58 and 1.98%, respectively), whereas participants from Ukraine and Russia had the highest (79.55 and 54.77%, respectively). In the analysis excluding participants from these four countries, the prevalence of DPN decreased slightly from the primary analysis (26.71–25.18%, Figure 2).

As shown in Table 1 and Supplementary Figure 1, women were more likely than men to have DPN (29.49 vs. 23.37%) in both the overall sample and in each age group. An increasing trend in prevalence of DPN with increasing age was observed and participants in the 56–60 years of age group had the highest prevalence, with 24.48% (95% CI: 19.86–29.78) in men and 30.75% (95% CI: 26.34–35.54) in women. In addition, those living in urban areas had a significantly higher prevalence of DPN than rural areas (28.75 vs. 14.72%, *p* < 0.05). However, excluding participants from those four countries modified the rural prevalence of DPN, which decreased dramatically in both male and female participants whereas urban prevalence of DPN increased slightly (Supplementary Table 2). Moreover, the gender-specific prevalence of DPN converged approximately to 25% in 56–60 and 61–65 years of age groups in the sensitivity analysis (Supplementary Figure 2).

**Table 1:** Prevalence and 95% CI of diabetic neuropathy by gender and living area.

	**Overall, %**	**Rural, %**	**Urban, %**
Both genders	26.71 (25.08–28.40)	14.72 (11.55–18.58)	28.75 (26.95–30.63)
Male	23.37 (21.09–25.81)	16.25 (11.29–22.82)	24.44 (21.97–27.10)
Female	29.49 (27.23–31.86)	13.68 (9.82–18.71)	32.46 (29.92–35.10)

A total of 2,135 (78.12%) participants had complete data for all variables of interest. Binary analyses results are presented in Table 2. Gender, residence, marital status, exercise, duration of diabetes, insulin use, hypertension, antihypertensive drugs use, cardiovascular disease, depressive symptoms, HbA1c, SBP, DBP, and BMI differed significantly between participants with and without DPN. The multilevel modeling results are shown in Table 3. In the multivariate analysis, duration of diabetes (OR: 1.08 per 1-year increase, 95% CI: 1.06–1.09), HbA1c (OR: 1.11 per 1% increase, 95% CI: 1.05–1.18), hypertension (OR: 1.58, 95% CI: 1.18–2.12), cardiovascular disease (OR: 2.07, 95% CI: 1.55–2.78), and depressive symptoms (OR: 1.92, 95% CI: 1.43–2.58) were independently and positively associated with DPN. Moreover, sensitivity analysis produced similar findings about risk factors of DPN between including and excluding participants from participants from Kenya (0.58%), Italy (1.98%), Russia (54.77%) and Ukraine (79.55%). The modified associations of duration of diabetes (OR: 1.07 per 1-year increase, 95% CI: 1.05–1.09), HbA1c (OR: 1.11 per 1% increase, 95% CI: 1.04–1.18), hypertension (OR: 1.40, 95% CI: 1.02–1.91), cardiovascular disease (OR: 2.08, 95% CI: 1.48–2.92) and depressive symptoms (OR: 1.85, 95% CI: 1.35–2.56) with DPN were similar to the estimates from the primary analysis in terms of trend and magnitude (Supplementary Table 3). In addition, risk factors for DPN in each country (except Italy, Kenya and Uganda due to scarce DPN patients) were also analyzed and presented in Supplementary Figures 3A–K. Specifically, there were country-dependent positive associations between the primary clinical correlates and the risks of DPN as follows: the duration of diabetes in six countries including China, Germany, India, Pakistan, Russia, and Serbia; the HbA1c concentrations in Germany and Serbia; hypertension in China; cardiovascular disease in China and Serbia; and depressive symptoms in Argentina and Mexico. Furthermore, the following factors decreased the risks of DPN in respective countries: higher level of education in Argentina, regular exercise in China, and being not married in Mexico. In contrast, the following factors potentiated the risk of DPN in respective countries: higher BMI in Poland and Russia, being older in Ukraine, and having regular family income in Bangladesh.

**Table 2:** Characteristics of the participants by diabetic neuropathy status.

	**Diabetic neuropathy**		
**Variable**	**No (*n* = 2003)**	**Yes (*n* = 730)**	***P***
Gender, *n* (%)			<0.001
Female	1,052 (52.52)	440 (60.27)	
Male	951 (47.48)	290 (39.73)	
Age, mean (SD), years	53.44 (9.31) (*n* = 2,000)	53.99 (8.71) (*n* = 729)	0.170
Location of Residence, *n* (%)			<0.001
Rural	336 (16.77)	58 (7.95)	
Urban	1,665 (83.13)	672 (92.05)	
Missing	2 (0.10)	0 (0.00)	
Marital status, *n* (%)			0.005
Married/cohabiting	1,482 (73.99)	500 (68.49)	
Single/widowed/divorced	518 (25.86)	228 (31.23)	
Missing	3 (0.15)	2 (0.27)	
Higher education, *n* (%)			0.280
No	1,370 (68.40)	515 (70.55)	
Yes	633 (31.60)	215 (29.45)	
Family income status, *n* (%)			0.500
No regular income	340 (16.97)	116 (15.89)	
Regular income	1,662 (82.98)	614 (84.11)	
Missing	1 (0.05)	0 (0.00)	
Smoking status, *n* (%)			0.520
Never	1,282 (64.00)	458 (62.74)	
Ever	438 (21.87)	175 (23.97)	
Current	274 (13.68)	96 (13.15)	
Missing	9 (0.45)	1 (0.14)	
Exercise (at least weekly), *n* (%)			<0.001
No	713 (35.60)	390 (53.42)	
Yes	1,274 (63.60)	338 (46.30)	
Missing	16 (0.80)	2 (0.27)	
Family history of diabetes, years			0.370
No	685 (34.20)	264 (36.16)	
Yes	1,312 (65.50)	466 (63.84)	
Missing	6 (0.30)	0 (0.00)	
Duration of diabetes, mean (SD)	8.05 (6.42) (*n* = 1,933)	10.91 (7.16) (*n* = 720)	<0.001
Insulin use, *n* (%)			<0.001
No	1,298 (64.80)	280 (38.36)	
Yes	699 (34.90)	449 (61.51)	
Missing	6 (0.30)	1 (0.14)	
HbA1c, mean (SD), %	7.97 (2.37) (*n* = 1,655)	8.70 (2.15) (*n* = 617)	<0.001
Hypertension, *n* (%)			<0.001
No	632 (31.55)	171 (23.42)	
Yes	1,371 (68.45)	559 (76.58)	
Antihypertensive drugs use, *n* (%)			<0.001
No	859 (42.89)	223 (30.55)	
Yes	1,118 (55.82)	493 (67.53)	
Missing	26 (1.30)	14 (1.92)	
SBP, mean (SD), mmHg	130.54 (17.87) (*n* = 1,944)	133.09 (18.70) (*n* = 708)	0.001
DBP, mean (SD), mmHg	79.38 (10.33) (*n* = 1,941)	80.82 (10.68) (*n* = 708)	0.002
BMI, mean (SD), kg/m^2^	28.63 (5.96) (*n* = 1,973)	29.43 (6.20) (*n* = 725)	0.002
Depressive symptoms, *n* (%)			<0.001
No	1,731 (86.42)	526 (72.05)	
Yes	259 (12.93)	200 (27.40)	
Missing	13 (0.65)	4 (0.55)	
Cardiovascular disease, *n* (%)			<0.001
No	1,710 (85.37)	481 (65.89)	
Yes	288 (14.38)	246 (33.70)	
Missing	5 (0.25)	3 (0.41)	

**Table 3:** Odds ratios for diabetic neuropathy determined by multilevel mixed-effects logistic regression.

	**Univariate analysis**		**Multivariate analysis**	
**Variable**	**Crude OR (95% CI)**	***P***	**Adjusted OR (95% CI)**	***P***
Age, per 5-year increase	1.15 (1.08–1.22)	<0.001	1.04 (0.96–1.13)	0.307
Gender (male vs. female)	0.79 (0.65–0.96)	0.017	0.85 (0.65–1.10)	0.214
Location of residence (urban vs. rural)	1.04 (0.74–1.46)	0.819	1.08 (0.70–1.65)	0.739
Marital status (not married vs. married)	1.16 (0.94–1.43)	0.175	0.94 (0.72–1.23)	0.653
Higher education (yes vs. no)	0.66 (0.53–0.82)	<0.001	0.77 (0.58–1.01)	0.062
Regular family income (yes vs. no)	0.88 (0.67–1.14)	0.330	1.26 (0.87–1.82)	0.217
Smoking status				
Ever vs. never	1.60 (1.25–2.04)	<0.001	1.34 (0.98–1.83)	0.069
Current vs. never	0.93 (0.69–1.26)	0.649	1.10 (0.76–1.59)	0.602
Higher exercise level (yes vs. no)	0.70 (0.57–0.86)	0.001	0.93 (0.72–1.20)	0.582
Family history of diabetes (yes vs. no)	1.12 (0.91–1.37)	0.291	0.96 (0.75–1.24)	0.759
Duration of diabetes, per 1-year increase	1.08 (1.07–1.10)	<0.001	1.08 (1.06–1.09)	<0.001
HbA_1_c, per 1% increase	1.17 (1.11–1.23)	<0.001	1.11 (1.05–1.18)	<0.001
Hypertension (yes vs. no)	2.13 (1.69–2.68)	<0.001	1.58 (1.18–2.12)	0.002
BMI, per 1 kg/m^2^ increase	1.02 (1.01–1.04)	0.006	1.02 (1.00–1.04)	0.125
Cardiovascular disease	3.00 (2.35–3.84)	<0.001	2.07 (1.55–2.78)	<0.001
Depressive symptoms (yes vs. no)	2.45 (1.93–3.11)	<0.001	1.92 (1.43–2.58)	<0.001

## Discussion

Our study indicates that the overall prevalence of DPN in this multi-ethnic cohort of adults with T2DM was 26.71%, and the independent and significant correlates of risk factors include duration of diabetes, poor glycemic control, and history of hypertension, cardiovascular disease and depressive symptoms. While the overall prevalence for DPN found in our study for adults with T2DM was similar to that reported previously (20), our estimates for prevalence of DPN in different countries varied substantially: ranging from 0.58% in Kenya to 79.55% in Ukraine. This large disparity could be attributed to different demography of the study population, cultural settings, and management of healthcare. We have also found that female diabetic patients were more likely to develop DPN than male patients (29.49 vs. 23.37%). This gender difference is consistent with a previous cross-sectional study (12).

Previous epidemiological studies have shown that the duration of diabetes and level of hyperglycemia are important risk factors for DPN (12, 13, 21). In addition, traditional cardiovascular risk factors (e.g., smoking, hypertension, dyslipidemia, and higher BMI) have also been reported to be associated with diabetes-related complications, including neuropathy (21, 22). Our study has not only confirmed these findings but also revealed depressive symptoms as a novel risk factor of DPN. Specifically, we have shown herein that T2DM patients with depressive symptoms doubled the risk of DPN, independent of a range of social-demographic factors, duration of diabetes, HbA1c, hypertension, BMI and cardiovascular disease.

Depressive symptoms are increasing rapidly worldwide among people with T2DM (23). Our previous study showed that the prevalence of moderate to severe levels of depression was 17.0% among those subjects (17). Diabetes can aggravate the symptoms of depression (24) and in turn depressive symptoms can also increase the risk of T2DM and complications (25). The recent Evaluation of Diabetes Treatment study has reported that people with pronounced depressive symptoms and high alcohol frequency had the highest risk of DPN (26). Indeed, a number of other studies have also demonstrated that poor glycemic control modifies the association between depressive symptoms and DPN among the T2DM population (27, 28). As mentioned above, the present study has illustrated that depressive symptoms were independently associated with an increased risk of DPN after adjusting the glycemic control status. A recent prospective cohort study with 2-year follow-up has also shown that depressive symptoms were both associated with poor glycemic control and incident macrovascular complications (29). However, our findings should be interpreted with caution due to the time lag between HbA1c measurement and identification of depressive symptoms.

Overall and country-specific analyses for risk factors of DPN in the present INTERPRET-DD study have presented us with a technical challenge. This was because the current collaborative study was carried out in 14 different countries with a large variation in the numbers of participants and cases of DPN among these countries. These variations might have been attributable to between-country characteristics and different cultural experiences of symptoms. Therefore, a sensitivity analysis was conducted to test the robustness of the overall analysis, the results of which supported that duration of diabetes, higher HbA1c, hypertension, and cardiovascular disease and depressive symptoms were strong independent risk factors of DPN. Furthermore, the duration of diabetes was the most common risk factor in six countries and overall analysis, followed by higher HbA1c, depressive symptoms, higher BMI, and cardiovascular disease as risk factors in two countries. Consistent with cross-sectional studies in China (30), Iran (10), Bangladesh (31) and 16 European countries (32), prolonged diabetes duration was significantly associated with higher risks of DPN. This suggests an urgent need for early screening and standardized management of DPN at a global scale. The identification of HbA1c, BMI, cardiovascular disease, and depressive symptoms as risk factors also points to the potential of blood glucose control, weight management, and prevention of dyslipidemia in decreasing DPN in specific countries. Thus, it is important to promote appropriate country-specific physical and psychological intervention strategies for T2DM-derived DPN.

Univariate analysis revealed that higher education level was significantly associated with lower risk of DPN, whereas smokers had elevated risks of DPN. Nevertheless, the significance of these two associations disappeared in multivariate analysis. Contrary to that, in the sensitivity analysis, the association between ever-smokers and risk of DPN remained in multivariate analysis. Therefore, there was variation in effects of education and smoking on the risk of DPN, as illustrated by the wider intervals of estimates in the multivariate analysis and the variation of findings in the sensitivity analysis. This variation in effects of education and smoking was likely due to the heterogeneous clinical profiles of participants in different countries and the facts that other risk factors might have attenuated their influence.

There are several limitations to our study. As this is a cross-sectional study, we are unable to identify the predictors but only the correlates of DPN. The limited numbers of participants in certain countries could have made the estimates of country-specific prevalence and risk factors of DPN questionable or not representative to those countries. Nevertheless, the current study also has several strengths. The INTERPRET-DD study was a collaborative study carried out simultaneously in 14 different countries. All collaborating researchers used the same protocol to collect baseline information, including the complete assessment of depressive symptoms using a clinically validated scale.

## Conclusion

The present study indicates that more than a quarter of T2DM individuals from 14 countries might develop DPN. The most significant, independent risk factors for the T2DM-associated DPN included duration of diabetes, poor glycemic control, and history of hypertension, cardiovascular disease and depressive symptoms. Our results have demonstrated a large variation among countries in the prevalence of the target disease and highlighted depressive symptoms as an important risk factor of DPN. Our findings will have significant implications in developing healthcare and treatment of T2DM complications.

## Data Availability Statement

The datasets generated for this study are available on request to the corresponding author.

## Ethics Statement

The studies involving human participants were reviewed and approved by Open University Research Ethics Committee and from each country's local ethics committee. The patients/participants provided their written informed consent to participate in this study.

## Author Contributions

ML, CL, and NS conceived and designed the study and reviewed the manuscript. YL analyzed the data and wrote the manuscript. PX, XC, DL, and RL helped to collected the data. All authors contributed to the article and approved the submitted version.

## Conflict of Interest

The authors declare that the research was conducted in the absence of any commercial or financial relationships that could be construed as a potential conflict of interest.

## Supplementary Material

Supplementary Figure 1 Prevalence of diabetic neuropathy by gender and age groups.

Supplementary Figure 2 Prevalence of diabetic neuropathy by gender and age groups after excluding participants from Kenya, Italy, Ukraine, and Russia.

Supplementary Figure 3 Risk factors for DPN in Argentina **(A)**, China **(B)**, Germany **(C)**, India **(D)**, Mexico **(E)**, Pakistan **(F)**, Poland **(G)**, Russia **(H)**, Serbia **(I)**, Ukraine **(J)**, and Bangladesh **(K)**. Red indicates significant associations with increased DPN risk and green indicates significant associations with decreased DPN risk.

Supplementary Table 1 Demographic profile of the study population by country.

Supplementary Table 2 Prevalence and 95% CI of diabetic neuropathy by gender and living area after excluding participants from Kenya, Italy, Ukraine and Russia.

Supplementary Table 3 Odds ratios for diabetic neuropathy, as determined by multilevel mixed-effects logistic regression in sensitivity analysis.
