# Supplementary figures and images for: Prevalence and Risk Factors for Diabetic Peripheral Neuropathy in Type 2 Diabetic Patients From 14 Countries: Estimates of the INTERPRET-DD Study
Source: Front Public Health. 2020 Oct 20;8:534372. doi: 10.3389/fpubh.2020.534372 (PMC7606804; doi:10.3389/fpubh.2020.534372)

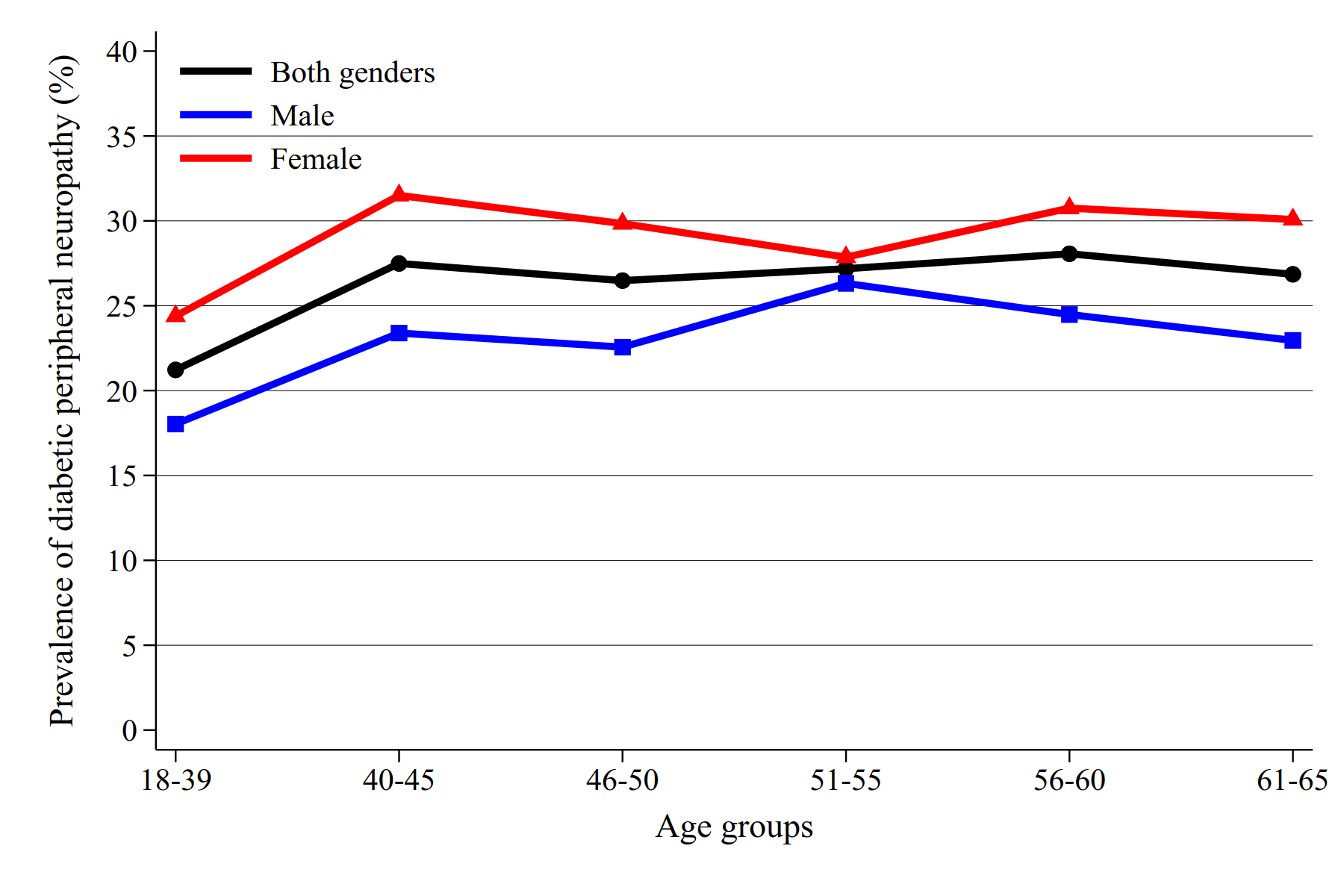

Supplement: Supplementary Figure 1 — Prevalence of diabetic neuropathy by gender and age groups. [file Image_1.TIF]

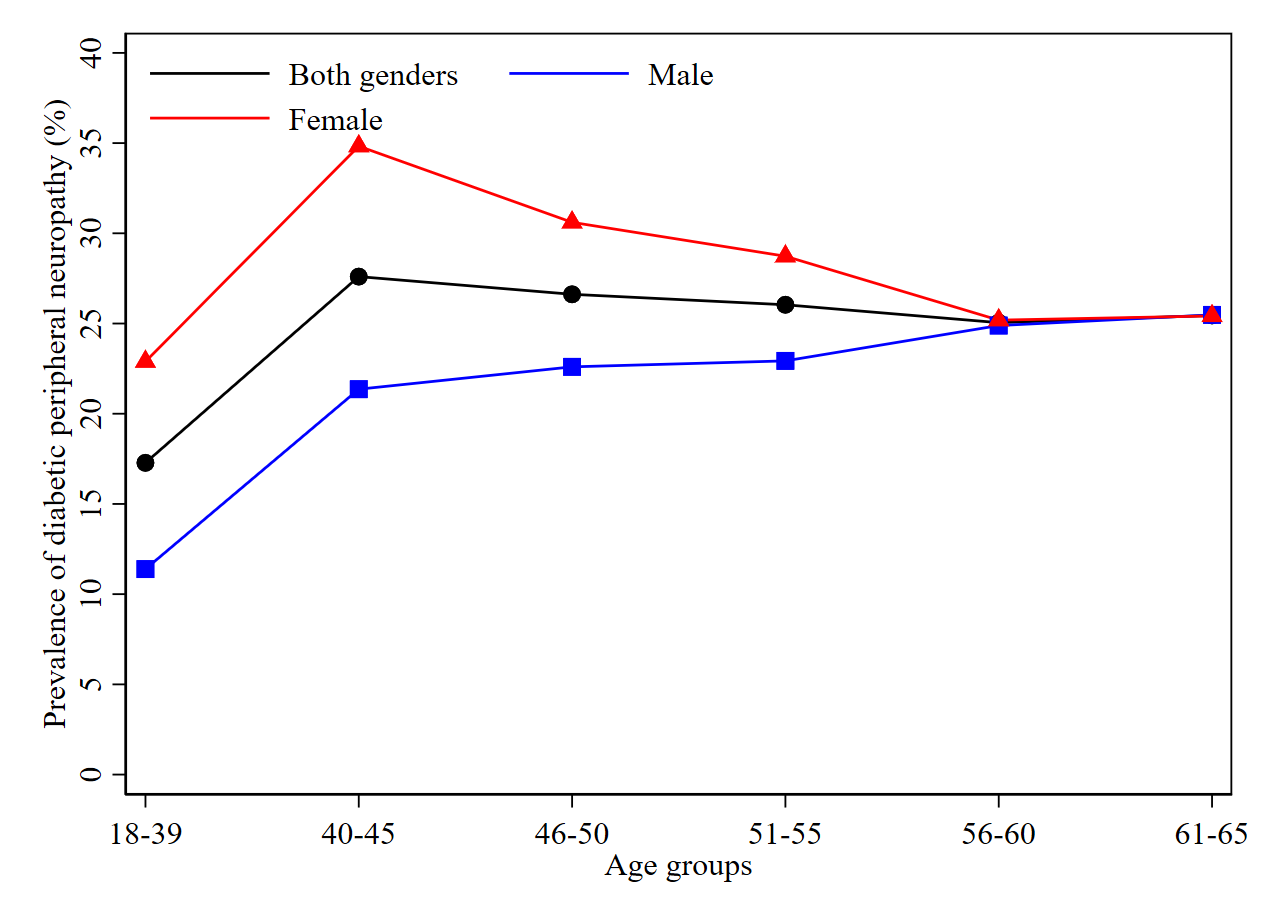

Supplement: Supplementary Figure 2 — Prevalence of diabetic neuropathy by gender and age groups after excluding participants from Kenya, Italy, Ukraine, and Russia. [file Image_2.TIF]
