# Supplementary material for: Prevalence and Risk Factors for Diabetic Peripheral Neuropathy in Type 2 Diabetic Patients From 14 Countries: Estimates of the INTERPRET-DD Study
Source: Front Public Health. 2020 Oct 20;8:534372. doi: 10.3389/fpubh.2020.534372 (PMC7606804; doi:10.3389/fpubh.2020.534372)
Supplement: Supplementary Table 1 — Demographic profile of the study population by country. [file Table_1.docx]

| Table S1. Demographic proﬁle of the study population by country | | | | | | | | | | | | | | |
| --- | --- | --- | --- | --- | --- | --- | --- | --- | --- | --- | --- | --- | --- | --- |
| Variable | Argentina | Bangladesh | China | Germany | India | Italy | Kenya | Mexico | Pakistan | Poland | Russia | Serbia | Ukraine | Uganda |
| Sample size, n | 134 | 194 | 388 | 116 | 188 | 202 | 171 | 203 | 196 | 213 | 199 | 200 | 132 | 197 |
| Gender, n (%) | | | | | | | | | | | | | |  |
| Female | 65 (48.51) | 99 (51.03) | 184 (47.42) | 42 (36.21) | 90 (47.87) | 84 (41.58) | 128 (74.85) | 129 (63.55) | 104 (53.06) | 100 (46.95) | 152 (76.38) | 113 (56.50) | 73 (55.30) | 129 (65.48) |
| Male | 69 (51.49) | 95 (48.97) | 204 (52.58) | 74 (63.79) | 98 (52.13) | 118 (58.42) | 43 (25.15) | 74 (36.45) | 92 (46.94) | 113 (53.05) | 47 (23.62) | 87 (43.50) | 59 (44.70) | 68 (34.52) |
| Age, mean (SD), years | 57.05 (7.39) (n=134) | 48.22 (9.09) (n=194) | 54.45 (8.95) (n=386) | 54.34 (7.47) (n=116) | 52.62 (9.64) (n=188) | 56.35 (7.17) (n=202) | 50.02 (10.95) (n=170) | 53.30 (8.21) (n=203) | 51.06 (8.83) (n=196) | 57.44 (7.36) (n=213) | 56.70 (7.33) (n=199) | 58.44 (5.71) (n=199) | 46.46 (12.04) (n=132) | 50.96 (8.27) (n=197) |
| Location of Residence, n (%) | | | | | | | | | | | | | |  |
| Rural | 2 (1.49) | 24 (12.37) | 33 (8.51) | 18 (15.52) | 31 (16.49) | 39 (19.31) | 143 (83.63) | 6 (2.96) | 31 (15.82) | 19 (8.92) | 14 (7.04) | 5 (2.50) | 0 (0.00) | 29 (14.72) |
| Urban | 130 (97.01) | 170 (87.63) | 355 (91.49) | 98 (84.48) | 157 (83.51) | 163 (80.69) | 28 (16.37) | 197 (97.04) | 165 (84.18) | 194 (91.08) | 185 (92.96) | 195 (97.50) | 132 (100.00) | 168 (85.28) |
| Missing | 2 (1.49) | 0 (0.00) | 0 (0.00) | 0 (0.00) | 0 (0.00) | 0 (0.00) | 0 (0.00) | 0 (0.00) | 0 (0.00) | 0 (0.00) | 0 (0.00) | 0 (0.00) | 0 (0.00) | 0 (0.00) |
| Marital status, n (%) |  |  |  |  |  |  |  |  |  |  |  |  |  |  |
| Married/co-habiting | 89 (66.42) | 153 (78.87) | 334 (86.08) | 69 (59.48) | 137 (72.87) | 146 (72.28) | 133 (77.78) | 118 (58.13) | 163 (83.16) | 157 (73.71) | 133 (66.83) | 155 (77.50) | 77 (58.33) | 118 (59.90) |
| Single/widowed/divorced | 44 (32.84) | 41 (21.13) | 54 (13.92) | 47 (40.52) | 50 (26.60) | 56 (27.72) | 38 (22.22) | 85 (41.87) | 33 (16.84) | 56 (26.29) | 66 (33.17) | 44 (22.00) | 55 (41.67) | 77 (39.09) |
| Missing | 1 (0.75) | 0 (0.00) | 0 (0.00) | 0 (0.00) | 1 (0.53) | 0 (0.00) | 0 (0.00) | 0 (0.00) | 0 (0.00) | 0 (0.00) | 0 (0.00) | 1 (0.50) | 0 (0.00) | 2 (1.02) |
| Higher education, n (%) | | | | | | | | | | | | | | |
| No | 86 (64.18) | 149 (76.80) | 214 (55.15) | 84 (72.41) | 156 (82.98) | 128 (63.37) | 157 (91.81) | 117 (57.64) | 167 (85.20) | 156 (73.24) | 85 (42.71) | 172 (86.00) | 71 (53.79) | 143 (72.59) |
| Yes | 48 (35.82) | 45 (23.20) | 174 (44.85) | 32 (27.59) | 32 (17.02) | 74 (36.63) | 14 (8.19) | 86 (42.36) | 29 (14.80) | 57 (26.76) | 114 (57.29) | 28 (14.00) | 61 (46.21) | 54 (27.41) |
| Family income status, n (%) | | | | | | | | | | | | | | |
| No regular income | 5 (3.73) | 47 (24.23) | 32 (8.25) | 14 (12.07) | 43 (22.87) | 30 (14.85) | 58 (33.92) | 97 (47.78) | 38 (19.39) | 16 (7.51) | 3 (1.51) | 19 (9.50) | 4 (3.03) | 50 (25.38) |
| Regular income | 129 (96.27) | 147 (75.77) | 356 (91.75) | 102 (87.93) | 145 (77.13) | 172 (85.15) | 112 (65.50) | 106 (52.22) | 158 (80.61) | 197 (92.49) | 196 (98.49) | 181 (90.50) | 128 (96.97) | 147 (74.62) |
| Missing | 0 (0.00) | 0 (0.00) | 0 (0.00) | 0 (0.00) | 0 (0.00) | 0 (0.00) | 1 (0.58) | 0 (0.00) | 0 (0.00) | 0 (0.00) | 0 (0.00) | 0 (0.00) | 0 (0.00) | 0 (0.00) |
| Smoking status, n (%) | | | | | | | | | | | | | | |
| Never | 54 (40.30) | 167 (86.08) | 241 (62.11) | 47 (40.52) | 138 (73.40) | 79 (39.11) | 156 (91.23) | 129 (63.55) | 161 (82.14) | 61 (28.64) | 153 (76.88) | 102 (51.00) | 71 (53.79) | 181 (91.88) |
| Ever | 54 (40.30) | 26 (13.40) | 72 (18.56) | 43 (37.07) | 40 (21.28) | 82 (40.59) | 13 (7.60) | 49 (24.14) | 18 (9.18) | 101 (47.42) | 23 (11.56) | 56 (28.00) | 23 (17.42) | 13 (6.60) |
| Current | 26 (19.40) | 0 (0.00) | 74 (19.07) | 25 (21.55) | 9 (4.79) | 38 (18.81) | 2 (1.17) | 25 (12.32) | 17 (8.67) | 50 (23.47) | 23 (11.56) | 41 (20.50) | 38 (28.79) | 2 (1.02) |
| Missing | 0 (0.00) | 1 (0.52) | 1 (0.26) | 1 (0.86) | 1 (0.53) | 3 (1.49) | 0 (0.00) | 0 (0.00) | 0 (0.00) | 1 (0.47) | 0 (0.00) | 1 (0.50) | 0 (0.00) | 1 (0.51) |
| Exercise (at least weekly), n (%) | | | | | | | | | | | | | |  |
| No | 50 (37.31) | 57 (29.38) | 104 (26.80) | 38 (32.76) | 67 (35.64) | 89 (44.06) | 45 (26.32) | 88 (43.35) | 112 (57.14) | 84 (39.44) | 167 (83.92) | 89 (44.50) | 92 (69.70) | 21 (10.66) |
| Yes | 83 (61.94) | 137 (70.62) | 281 (72.42) | 72 (62.07) | 120 (63.83) | 109 (53.96) | 125 (73.10) | 115 (56.65) | 83 (42.35) | 129 (60.56) | 32 (16.08) | 110 (55.00) | 40 (30.30) | 176 (89.34) |
| Missing | 1 (0.75) | 0 (0.00) | 3 (0.77) | 6 (5.17) | 1 (0.53) | 4 (1.98) | 1 (0.58) | 0 (0.00) | 1 (0.51) | 0 (0.00) | 0 (0.00) | 1 (0.50) | 0 (0.00) | 0 (0.00) |
| Family history of diabetes, years | | | | | | | | | | | | | | |
| No | 41 (30.60) | 90 (46.39) | 128 (32.99) | 36 (31.03) | 55 (29.26) | 33 (16.34) | 91 (53.22) | 23 (11.33) | 69 (35.20) | 68 (31.92) | 96 (48.24) | 59 (29.50) | 70 (53.03) | 90 (45.69) |
| Yes | 92 (68.66) | 104 (53.61) | 258 (66.49) | 79 (68.10) | 132 (70.21) | 168 (83.17) | 80 (46.78) | 180 (88.67) | 127 (64.80) | 145 (68.08) | 103 (51.76) | 141 (70.50) | 62 (46.97) | 107 (54.31) |
| Missing | 1 (0.75) | 0 (0.00) | 2 (0.52) | 1 (0.86) | 1 (0.53) | 1 (0.50) | 0 (0.00) | 0 (0.00) | 0 (0.00) | 0 (0.00) | 0 (0.00) | 0 (0.00) | 0 (0.00) | 0 (0.00) |
| Duration of diabetes, mean (SD) | 9.74 (6.39) (n=131) | 7.21 (5.19) (n=194) | 9.27 (6.97) (n=315) | 8.06 (8.90) (n=116) | 8.52 (6.67) (n=188) | 9.88 (7.54) (n=201) | 7.13 (6.06) (n=171) | 11.26 (7.91) (n=203) | 8.43 (6.11) (n=196) | 9.46 (7.06) (n=213) | 8.86 (6.15) (n=199) | 9.31 (5.99) (n=198) | 8.77 (6.55) (n=132) | 6.92 (5.46) (n=196) |
| Insulin use, n (%) | | | | | | | | | | | | | | |
| No | 92 (68.66) | 86 (44.33) | 201 (51.80) | 73 (62.93) | 151 (80.32) | 147 (72.77) | 98 (57.31) | 97 (47.78) | 126 (64.29) | 147 (69.01) | 89 (44.72) | 89 (44.50) | 39 (29.55) | 143 (72.59) |
| Yes | 40 (29.85) | 108 (55.67) | 187 (48.20) | 43 (37.07) | 36 (19.15) | 54 (26.73) | 71 (41.52) | 106 (52.22) | 70 (35.71) | 65 (30.52) | 110 (55.28) | 111 (55.50) | 93 (70.45) | 54 (27.41) |
| Missing | 2 (1.49) | 0 (0.00) | 0 (0.00) | 0 (0.00) | 1 (0.53) | 1 (0.50) | 2 (1.17) | 0 (0.00) | 0 (0.00) | 1 (0.47) | 0 (0.00) | 0 (0.00) | 0 (0.00) | 0 (0.00) |
| HbA1c, mean (SD), % | 7.72 (1.76) (n=132) | 8.33 (1.75) (n=103) | 7.74 (2.02) (n=330) | 6.89 (1.26) (n=110) | 8.77 (2.07) (n=186) | 7.13 (1.11) (n=199) | 10.11 (4.16) (n=170) | 8.60 (2.42) (n=99) | 8.23 (2.06) (n=196) | 6.91 (1.22) (n=194) | 8.29 (2.10) (n=199) | 8.52 (1.93) (n=197) | 9.16 (1.82) (n=126) | 10.22 (4.82) (n=31) |
| Hypertension, n (%) | | | | | | | | | | | | | |  |
| No | 42 (31.34) | 90 (46.39) | 156 (40.21) | 15 (12.93) | 60 (31.91) | 42 (20.79) | 42 (24.56) | 70 (34.48) | 103 (52.55) | 20 (9.39) | 30 (15.08) | 35 (17.50) | 56 (42.42) | 42 (21.32) |
| Yes | 92 (68.66) | 104 (53.61) | 232 (59.79) | 101 (87.07) | 128 (68.09) | 160 (79.21) | 129 (75.44) | 133 (65.52) | 93 (47.45) | 193 (90.61) | 169 (84.92) | 165 (82.50) | 76 (57.58) | 155 (78.68) |
| Antihypertensive drugs use, n (%) | | | | | | | | | | | | | | |
| No | 53 (39.55) | 90 (46.39) | 208 (53.61) | 35 (30.17) | 74 (39.36) | 67 (33.17) | 71 (41.52) | 101 (49.75) | 116 (59.18) | 43 (20.19) | 51 (25.63) | 38 (19.00) | 67 (50.76) | 68 (34.52) |
| Yes | 81 (60.45) | 72 (37.11) | 176 (45.36) | 81 (69.83) | 113 (60.11) | 135 (66.83) | 100 (58.48) | 101 (49.75) | 80 (40.82) | 170 (79.81) | 148 (74.37) | 160 (80.00) | 65 (49.24) | 129 (65.48) |
| Missing | 0 (0.00) | 32 (16.49) | 4 (1.03) | 0 (0.00) | 1 (0.53) | 0 (0.00) | 0 (0.00) | 1 (0.49) | 0 (0.00) | 0 (0.00) | 0 (0.00) | 2 (1.00) | 0 (0.00) | 0 (0.00) |
| SBP, mean (SD), mmHg | 125.98 (12.63) (n=131) | 123.92 (11.85) (n=194) | 126.17 (14.75) (n=378) | 138.70 (18.26) (n=113) | 133.29 (16.12) (n=187) | 129.55 (15.69) (n=202) | 137.39 (25.61) (n=171) | 119.61 (17.05) (n=148) | 131.20 (17.71) (n=196) | 139.27 (18.71) (n=204) | 134.69 (15.79) (n=199) | 134.18 (15.69) (n=200) | 131.49 (20.81) (n=132) | 135.41 (20.95) (n=197) |
| DBP, mean (SD), mmHg | 78.47 (8.41) (n=130) | 78.71 (6.55) (n=194) | 79.72 (9.69) (n=378) | 82.80 (11.04) (n=113) | 75.43 (9.84) (n=187) | 79.85 (9.17) (n=200) | 77.20 (13.40) (n=171) | 75.82 (8.86) (n=148) | 81.40 (10.14) (n=196) | 80.17 (9.72) (n=204) | 82.65 (8.93) (n=199) | 83.79 (9.31) (n=200) | 74.83 (10.40) (n=132) | 83.50 (14.16) (n=197) |
| BMI, mean (SD), kg/m^2^ | 32.68 (6.26) (n=128) | 25.67 (3.86) (n=192) | 25.73 (3.73) (n=388) | 33.37 (7.82) (n=116) | 26.95 (5.25) (n=188) | 31.12 (6.25) (n=200) | 27.92 (6.43) (n=167) | 28.84 (5.31) (n=184) | 27.32 (4.74) (n=196) | 30.81 (5.86) (n=211) | 33.59 (6.92) (n=199) | 29.19 (5.29) (n=200) | 28.03 (4.42) (n=132) | 27.98 (5.60) (n=197) |
| Depressive symptoms, n (%) | | | | | | | | | | | | | |  |
| No | 116 (86.57) | 131 (67.53) | 330 (85.05) | 84 (72.41) | 158 (84.04) | 175 (86.63) | 150 (87.72) | 142 (69.95) | 160 (81.63) | 183 (85.92) | 152 (76.38) | 173 (86.50) | 108 (81.82) | 195 (98.98) |
| Yes | 17 (12.69) | 63 (32.47) | 55 (14.18) | 19 (16.38) | 30 (15.96) | 27 (13.37) | 21 (12.28) | 61 (30.05) | 36 (18.37) | 30 (14.08) | 47 (23.62) | 27 (13.50) | 24 (18.18) | 2 (1.02) |
| Missing | 1 (0.75) | 0 (0.00) | 3 (0.77) | 13 (11.21) | 0 (0.00) | 0 (0.00) | 0 (0.00) | 0 (0.00) | 0 (0.00) | 0 (0.00) | 0 (0.00) | 0 (0.00) | 0 (0.00) | 0 (0.00) |
| Cardiovascular disease, n (%) | | | | | | | | | | | | | | |
| No | 120 (89.55) | 189 (97.42) | 290 (74.74) | 87 (75.00) | 178 (94.68) | 164 (81.19) | 164 (95.91) | 187 (92.12) | 169 (86.22) | 127 (59.62) | 124 (62.31) | 147 (73.50) | 61 (46.21) | 184 (93.40) |
| Yes | 14 (10.45) | 5 (2.58) | 92 (23.71) | 29 (25.00) | 9 (4.79) | 38 (18.81) | 7 (4.09) | 16 (7.88) | 27 (13.78) | 86 (40.38) | 75 (37.69) | 53 (26.50) | 71 (53.79) | 12 (6.09) |
| Missing | 0 (0.00) | 0 (0.00) | 6 (1.55) | 0 (0.00) | 1 (0.53) | 0 (0.00) | 0 (0.00) | 0 (0.00) | 0 (0.00) | 0 (0.00) | 0 (0.00) | 0 (0.00) | 0 (0.00) | 1 (0.51) |
