# Supplementary material for: Prevalence and Risk Factors for Diabetic Peripheral Neuropathy in Type 2 Diabetic Patients From 14 Countries: Estimates of the INTERPRET-DD Study
Source: Front Public Health. 2020 Oct 20;8:534372. doi: 10.3389/fpubh.2020.534372 (PMC7606804; doi:10.3389/fpubh.2020.534372)
Supplement: Supplementary Table 2 — Prevalence and 95% CI of diabetic neuropathy by gender and living area after excluding participants from Kenya, Italy, Ukraine and Russia. [file Table_2.docx]

| **Supplementary materials**  Table S2. Prevalence and 95% CI of diabetic neuropathy by gender and living area after excluding participants from Kenya, Italy, Ukraine and Russia | | | |
| --- | --- | --- | --- |
|  | Overall, % | Rural, % | Urban, % |
| Both genders | 25.18 (25.08-27.12) | 25.76 (20.14-32.30) | 25.15 (23.21-27.19) |
| Male | 22.90 (20.36-25.64) | 25.51 (17.86-35.04) | 22.63 (19.98-25.52) |
| Female | 27.30 (24.69-30.07) | 26.00 (18.35-35.46) | 27.46 (24.72-30.39) |

| Table S3. Odds ratios for diabetic neuropathy, as determined by multilevel mixed-effects logistic regression in sensitivity analysis | | | | | |
| --- | --- | --- | --- | --- | --- |
|  | Univariate analysis | |  | Multivariate analysis | |
| Variable | Crude OR (95% CI) | *P* |  | Adjusted OR (95% CI) | *P* |
| Age, per 5-year increase | 1.02 (1.00-1.03) | 0.011 |  | 1.00 (0.98-1.01) | 0.732 |
| Gender (male vs female) | 0.83 (0.67-1.02) | 0.080 |  | 0.78 (0.58-1.05) | 0.100 |
| Location of residence (urban vs rural) | 0.93 (0.65-1.32) | 0.686 |  | 0.95 (0.60-1.51) | 0.824 |
| Marital status (not married vs married) | 1.17 (0.92-1.48) | 0.207 |  | 0.97 (0.71-1.32) | 0.839 |
| Higher education (yes vs no) | 0.69 (0.54-0.89) | 0.004 |  | 0.79 (0.57-1.09) | 0.148 |
| Regular family income (yes vs no) | 0.89 (0.68-1.17) | 0.398 |  | 1.26 (0.86-1.84) | 0.233 |
| Smoking status |  |  |  |  |  |
| Ever vs never | 1.87 (1.44-2.43) | <0.001 |  | 1.56 (1.11-2.21) | 0.011 |
| Current vs never | 1.22 (0.87-1.71) | 0.252 |  | 1.36 (0.90-2.06) | 0.144 |
| Higher exercise level (yes vs no) | 0.67 (0.54-0.83) | <0.001 |  | 0.91 (0.69-1.20) | 0.501 |
| Family history of diabetes (yes vs no) | 1.16 (0.92-1.46) | 0.202 |  | 0.96 (0.72-1.28) | 0.768 |
| Duration of diabetes, per 1-year increase | 1.07 (1.06-1.09) | <0.001 |  | 1.07 (1.05-1.09) | <0.001 |
| HbA_1_c, per 1% increase | 1.16 (1.10-1.23) | <0.001 |  | 1.11 (1.04-1.18) | <0.001 |
| Hypertension (yes vs no) | 1.72 (1.35-2.20) | <0.001 |  | 1.40 (1.02-1.91) | 0.035 |
| BMI, per 1 kg/m^2^ increase | 1.01 (0.99-1.03) | 0.296 |  | 1.00 (0.98-1.03) | 0.731 |
| Cardiovascular disease | 2.75 (2.07-3.64) | <0.001 |  | 2.08 (1.48-2.92) | <0.001 |
| Depressive symptoms (yes vs no) | 2.38 (1.84-3.09) | <0.001 |  | 1.85 (1.35-2.56) | <0.001 |
